# Proteomic and Transcriptomic Analyses Indicate Metabolic Changes and Reduced Defense Responses in Mycorrhizal Roots of *Oeceoclades maculata* (Orchidaceae) Collected in Nature

**DOI:** 10.3390/jof6030148

**Published:** 2020-08-26

**Authors:** Rafael B. S. Valadares, Silvia Perotto, Adriano R. Lucheta, Eder C. Santos, Renato M. Oliveira, Marcio R. Lambais

**Affiliations:** 1 Escola Superior de Agricultura “Luiz de Queiroz”, Depto de Ciência do Solo, Universidade de São Paulo, Av. Pádua Dias 11, Piracicaba 13418-900, Brazil; mlambais@usp.br; 2 Instituto Tecnológico Vale. Rua Boaventura da Silva 955, Belém 66050-000, Brazil; renato.oliveira@pq.itv.org; 3 Dipartimento di Scienze della Vita e Biologia dei Sistemi, Università di Torino e IPSP-CNR, Viale Mattioli 25, 10125 Torino, Italy; 4 SENAI Innovation Institute for Mineral Technologies, Avenida Brás de Aguiar, 548, Belém 66035-405, Brazil; adriano.isi@senaipa.org.br; 5 Universidade Tecnológica Federal do Paraná, Linha Santa Bárbara, Francisco Beltrão 85601-970, Brazil; edersantos@utfpr.edu.br; 6 Instituto de Ciências Biológicas, Universidade Federal de Minas Gerais, Av. Pres. Antônio Carlos, 6627, Belo Horizonte 31270-901, Brazil

**Keywords:** orchid mycorrhiza, proteome, transcriptome, plant defense responses, nitrogen metabolism, hormones

## Abstract

Orchids form endomycorrhizal associations with fungi mainly belonging to basidiomycetes. The molecular events taking place in orchid mycorrhiza are poorly understood, although the cellular changes necessary to accommodate the fungus and to control nutrient exchanges imply a modulation of gene expression. Here, we used proteomics and transcriptomics to identify changes in the steady-state levels of proteins and transcripts in the roots of the green terrestrial orchid *Oeceoclades maculata*. When mycorrhizal and non-mycorrhizal roots from the same individuals were compared, 94 proteins showed differential accumulation using the label-free protein quantitation approach, 86 using isobaric tagging and 60 using 2D-differential electrophoresis. After *de novo* assembly of transcriptomic data, 11,179 plant transcripts were found to be differentially expressed, and 2175 were successfully annotated. The annotated plant transcripts allowed the identification of up- and down-regulated metabolic pathways. Overall, proteomics and transcriptomics revealed, in mycorrhizal roots, increased levels of transcription factors and nutrient transporters, as well as ethylene-related proteins. The expression pattern of proteins and transcripts involved in plant defense responses suggested that plant defense was reduced in *O. maculata* mycorrhizal roots sampled in nature. These results expand our current knowledge towards a better understanding of the orchid mycorrhizal symbiosis in adult plants under natural conditions.

## 1. Introduction

Orchidaceae is the largest flowering plant family and accounts for approximately 10% of the global plant diversity, with more than 28,000 described species [1]. Orchids produce endosperm-lacking seeds that are highly dependent on mycorrhizal fungi for germination and subsequent development [2]. After germination, orchid seeds develop into protocorms [3], heterotrophic structures that receive organic carbon and other nutrients from the mycorrhizal symbiont. This fungus-dependent trophic strategy is known as mycoheterotrophy [4,5]. Most orchid species later produce green leaves and become photoautotrophic, although mycoheterotrophy is retained throughout the life history by non-photosynthetic orchids and green orchids living in shaded environments [6]. Unlike other mycorrhizal types, nutrient and carbon transfer in orchid mycorrhiza (OM) seems to favor the plant over the fungus, especially during the mycoheterotrophic stages. For this reason, the OM relationship has been described by some authors as an example of reverse parasitism [3,6], even though the mycorrhizal association has been found to be mutualistic in the adult green-leaved terrestrial orchid *Goodyera repens*, with a net transfer of photosynthesis-derived carbon from the plant to the fungus [7,8].

The molecular mechanisms modulating OM are starting to come to light, especially during seed germination and protocorm development (see [9]). Suppression subtractive hybridization (SSH) was used to identify genes differentially expressed in symbiotically germinated *Dendrobium officinale* seeds, as compared to non-germinated seeds [10], and revealed up-regulation of general defense responses, cell wall modification, reactive oxygen species detoxification, secondary metabolites, and changes in hormone balance. More recently, proteomics and transcriptomics were used to compare symbiotic and asymbiotic *D. officinale* protocorms [11] and confirmed the previous results on this orchid species. A 2D-gel based approach suggests that protocorm development of *Oncidium sphacelatum* was enhanced by a mycorrhizal *Thanatephorus* sp. isolate through processes involving cell cycle proteins, purine recycling, ribosome biogenesis, and changes in energy metabolism [12]. A significant increase in antioxidant metabolism and nutrient transport was found during the development of *O. sphacelatum* protocorms in the presence of *Ceratobasidium* [13]. More recently, investigations on *Serapias vomeracea* protocorms colonized by *Tulasnella calospora* showed that expression of plant and fungal genes involved in nitrogen metabolism changed in symbiosis [14].

Most molecular studies of OM have focused on the protocorm stage, which can be obtained in vitro for many orchid species. By contrast, far less is known on the mycorrhizal interactions in the roots of adult orchids. The first studies on gene expression in OM roots showed up-regulation of a nucleotide-binding protein and down-regulation of a trehalose-phosphate phosphatase in mycorrhizal roots of *Cypripedium parviflorum* var. *pubescens* [15]. More recently, comparison of gene expression in mycorrhizal roots from green and albino individuals of *Epipactis helleborine* showed, in the albino plants, the up-regulation of antioxidant metabolism and transport of some nutrients [16].

**Figure 1:**
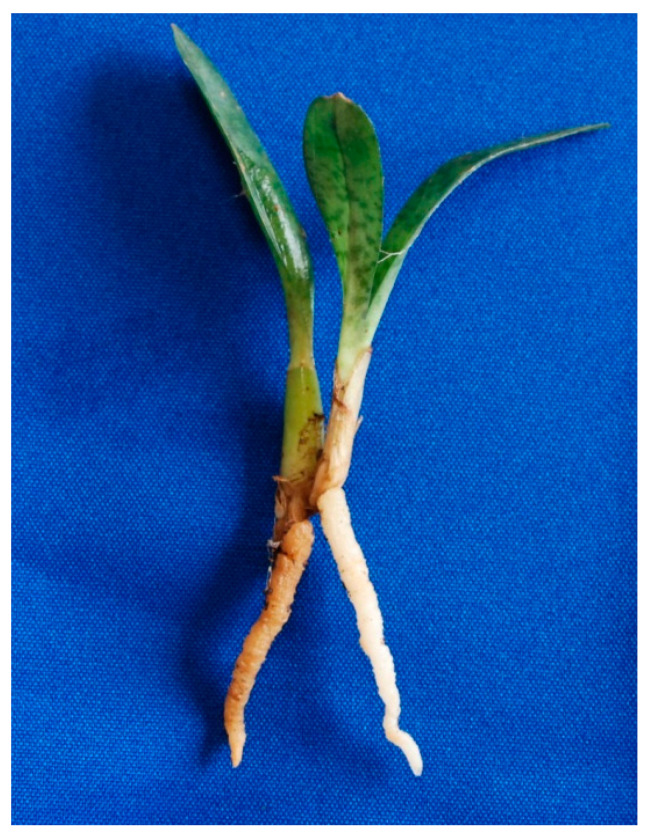
A plant of *Oeceoclades maculata* showing an older and darker mycorrhizal root on the left and a younger and paler non-mycorrhizal root on the right. Roots belonging to the two types were checked for their mycorrhizal status by microscopic screening before being subject to molecular analyses.

Here, we applied different proteomic techniques, as well as transcriptomics, to investigate plant proteins and genes differentially expressed in mycorrhizal and non-mycorrhizal roots of adult plants of *Oeceoclades maculata* (Lindley) Lindley. This terrestrial orchid, first described in a Brazilian orchid collection in 1827, has since spread at an accelerated rate through tropical and subtropical areas [17]. Individuals of *O. maculata* have, in general, one or two fully colonized darker roots, whereas the younger and paler roots are not colonized and accumulate large amounts of starch (Figure 1). The possibility to analyze mycorrhizal and non-mycorrhizal roots from the same plant individuals makes *O. maculata* an interesting experimental system to investigate the local molecular changes occurring during OM interactions. In mycorrhizal roots, transcriptomic and proteomic data revealed increased levels of transcription factors, sugar and amino acid transporters, and ethylene-related proteins. By contrast, proteins and transcripts involved in plant defense responses, including chitinase and a mannose-binding lectin, were reduced in mycorrhizal roots, suggesting that the presence of the fungal symbiont, identified as *Psathyrella candolleana* (Basidiomycota), leads to local modulation of plant defense responses in the orchid tissues.

## 2. Materials and Methods

### 2.1. Root Sampling

Adult healthy plants of *O. maculata* of about the same size were harvested in the dry season in a semideciduous forest plot (24 × 4 m) in the Piracicaba campus of the University of Sao Paulo (Sao Paulo, Brazil). For each plant, roots were excised using a sterile scalpel and washed with distilled water. Roots were then screened by microscopy for the presence or absence of mycorrhizal fungi. After screening, root samples were placed in microcentrifuge tubes, immediately frozen in liquid nitrogen, and stored at −80 °C. Mycorrhizal and non-mycorrhizal root fragments from four *O. maculata* plants were pooled to make a biological replicate of mycorrhizal (Myc) and non-mycorrhizal (Non-Myc) root samples, respectively. Three biological replicates of Myc and Non-Myc root samples were used for the proteomic analyses (12 plants in total). For the transcriptome, 12 additional plants were harvested in the same season and in the same forest plot, following the same experimental design (3 biological replicates, each consisting of a pool of roots from 4 adult plants).

### 2.2. Fungal Identification

Highly colonized *O. maculata* root fragments were selected under a Zeiss (Jena, Germany) stereomicroscope at 10–40× magnification. After removal of the outer root layers, root segments containing fungal pelotons were frozen in liquid nitrogen and grounded to a fine powder. DNA was extracted from 500 mg of grounded tissue using the Fast DNA Spin kit (MP Bio, Santa Ana, CA, USA) following the manufacturer’s instructions. The internal transcribed spacer (ITS) region of fungal ribosomal DNA (rDNA) was amplified by polymerase chain reaction (PCR) using the primers ITS1F and ITS4 [18] and total DNA as a template. Unless otherwise stated, reagents were obtained from Life Technologies (Carlsbad, CA, USA). PCR amplification was performed in 25 µL of a solution containing 1 × Taq Polymerase Buffer, 1.5 mM MgCl_2_, 0.2 mM dNTPs, 1.5 U of Recombinant Taq DNA Polymerase, 25 pmol of each primer, and 100 ng of template DNA. The amplification conditions were initial denaturing at 95 °C for 5 min, followed by 30 cycles of 94 °C for 1 min, 56 °C for 45 s, and 72 °C for 2 min, and a final extension for 10 min at 72 °C. Amplicons of about 600 bp were excised from 1% agarose gels after electrophoresis and purified. Purified amplicons were cloned into a pGEMT-Easy plasmid (Promega, Fitchburg, WI, USA), and plasmid transformed into *Escherichia coli* (DH5-α) competent cells by heat-shock. Bacterial cells containing recombinant plasmids were selected in solid LB media containing ampicillin (100 µg mL^−1^) and X-Gal (5-Brome-4-chloro-3-indolyl-b-D-galactoside) and transferred to fresh liquid LB media plus ampicillin (100 µg mL^−1^). Bacterial cells were grown overnight at 37 °C, and plasmids were extracted by alkaline lyses [19].

A total of 96 clones were sequenced by the Sanger method using an ABI 3100 Automatic Sequencer (Applied Biosystems, Foster City, CA, USA), BigDye Terminator V 3.1 Cycle Sequencing Kit, and T7 primer (5′-TAA TAC GAC TCA CTA TAG GGC-3′), following the manufacturer’s instructions. DNA sequences were filtered by quality (quality parameter >20) using Phred software (Univerisity of Washignton, Forks, WA, USA) [20] and aligned using BioEdit 7.2.5 software (Ibis Bioscience, Carlsbad, CA, USA). All the polymorphic bases in the alignment were checked manually in the chromatograms to ensure base calling. The sequences were compared against the National Center for Biotechnology Information (NCBI) nucleotide database (NT/NR) for taxonomical affiliation using BLAST [21]. A Neighbor-joining phylogenetic tree (Jukes-Cantor algorithm and 999 replicates bootstrap test) was constructed with the Mega 6 software [22] using the ITS sequences obtained from *O. maculata*, ITS sequences of the closest relatives based on Blast searches, and ITS sequences of some common orchid mycorrhizal isolates.

### 2.3. Protein Extraction

Proteins were extracted following the procedure described by [23] for recalcitrant plant tissues, with some modifications. Roots were ground to a fine powder in liquid nitrogen using a pestle and mortar. The powdered tissue (200 µg) was placed in microcentrifuge tubes and resuspended in 1.5 mL cold acetone containing 1% PMSF, 2% *β*-mercaptoethanol, and 1% polyvinylpolypyrrolidone (PVPP). After sonication (5 times for 20 s on ice), the tubes were centrifuged at 10,000 *g* for 3 min at 4 °C. The pellet was washed twice with cold acetone and three times with cold 10% trichloroacetic acid (TCA) in acetone or until it was colorless, then twice with cold aqueous 10% TCA, and finally twice with cold 80% acetone. After each washing, the pellet was resuspended completely by vortexing and then centrifuged as above. The final pellet was dried at room temperature and used for phenolic protein extraction. Dried pellet was resuspended in 800 µL phenol (Tris-HCl, pH 8.0) and 800 µL SDS buffer (30% sucrose, 2% SDS, 0.1 M Tris-HCl, pH 8.0, 5% *β*-mercaptoethanol) in a 2.0 mL microcentrifuge tube. The mixture was vortexed thoroughly for 30 s, and the phenol phase was separated by centrifugation at 10,000× *g* for 3 min at 4 °C. The upper phenol phase was transferred to fresh microcentrifuge tubes. Five volumes of 0.1 M ammonium acetate in cold methanol were added to the phenol phase, and the proteins precipitated at −80 °C for 30 min. Precipitated proteins were recovered after centrifugation at 10,000× *g* for 5 min at 4 °C and washed with cold 80% acetone twice and with 70% ethanol. The final pellet was dried, and proteins dissolved in a detergent-free buffer (7 M urea, 2 M thiourea) for mass spectrometry or 2-D electrophoresis rehydration buffer (8 M urea, 4% CHAPS, 2% IPG buffer, 20 mM dithiothreitol) for gel electrophoresis.

### 2.4. Protein Sample Preparation for 2D-Differential Electrophoresis

For the analytical electrophoresis, an aliquot of protein solution containing 100 µg of protein was used for labeling with CyDye™ Fluor minimal labeling reagents (Lumiprobe, Hallandale Beach, FL, USA). Proteins were solubilized in a minimum volume of CyDye labeling-compatible lysis buffer containing 40 mM Tris–HCl, 8 M urea, 4% (*w*/*v*) CHAPS (pH 8.5). Proteins from each biological replicate were labeled as following: gels 1 and 3: Cy3—mycorrhizal (Myc) and Cy5—non-mycorrhizal (Non-Myc) roots; gel 2: Cy3—Non-Myc and Cy5—Myc. Labeling reactions were performed according to the two dimensional difference gel electrophoresis 2D-DIGE minimal labeling protocol. The labeled samples for each experiment were mixed together, and the volume adjusted to 340 µL with rehydration buffer to a final solution containing 8 M urea, 2% (*w*/*v*) CHAPS, 0.5% DTT, 0.5% IPG buffer 4–7 (GE Healthcare, São Paulo, Brazil).

To identify proteins of interest by mass spectrometry (Maldi TOF-TOF), one additional preparative gel for each treatment (Myc and Non-Myc) was prepared as described above, except that 250 µg of protein was used.

Samples prepared for analytical and preparative electrophoresis were submitted to isoelectric focusing (IEF) and 2D gel electrophoresis in a single run using an Etan Dalt Six electrophoresis system (GE Healthcare, Sao Paulo, Brazil). Samples were added to IPG immobiline dry strips pH 4–7 (GE Healthcare, São Paulo, Brazil) and rehydrated for 12 h. Voltage settings for IEF were 250 V for 4 h; 300 V, 500 V, 1000 V, 3000 V, and 5000 V for 1 h each; 8000 V until achieved a total of 40 kVh. For the second dimension, the gel strips were incubated with equilibration buffer 1 (50 mM Tris-HCl pH 8.8, 6 M urea, 30% glycerol, 2% SDS, 0.002% bromophenol blue, 1% DTT) and equilibration buffer 2 (50 mM Tris-HCl pH 8.8, 6 M urea, 30% glycerol, 2% SDS, 0.002% bromophenol blue, 2.5% iodoacetamide) for 30 min each and, subsequently, placed onto 12.5% polyacrylamide gel (26 × 32 cm) with a Tris-glycine buffer system, as described by [24]. Strips were overlaid with an agarose sealing solution (0.25 M Tris-HCl, 1.92 M glycine, 1% SDS, 0.5% agarose, 0.002% bromophenol blue). The initial 2D electrophoresis setting was 5 W (constant and maximal 20 mA), followed by a 6 h at 30 W per gel (constant and maximal 50 mA). The two preparative gels were fixed overnight in 40% methanol and 10% acetic acid and then washed three times for 30 min with distilled water prior to staining with 0.1% Coomassie brilliant blue CBB-G250 for 48 h. Analytical gels were scanned on a Typhoon Trio Fluorescence Scanner (GE Healthcare, São Paulo, Brazil) at 300-micron resolution using excitation wavelengths of 532 nm for Cy3™ and 633 nm for Cy5™.

Gel analysis of the multiplexed 2D gel images was performed with the Progenesis SameSpots V 4.5 software (NonLinear Dynamics, New Castle, UK). Spot volumes were normalized by a ratiometric approach to center the standardized abundance values to one, where standardized abundance is the Cy3 normalized spot volume divided by the Cy5 normalized spot volume. Spots that were significantly different between treatments (ANOVA, *p* < 0.05) and the fold change >1.5 were selected for further analyses.

Protein spots were manually excised from gels and placed in 1.5 mL microcentrifuge tubes, destained with 50% acetonitrile (ACN) and 25 mM ammonium bicarbonate. Gel plugs were dehydrated with 100% ACN and rehydrated with 20 mM DTT; after incubating for 40 min at 56 °C, the supernatant was discarded and replaced with 55 mM iodoacetamide. The tubes were stored in the dark for 30 min, and the gel pieces were dehydrated again with 100% ACN and allowed to air dry after solvent removal. The gel pieces were rehydrated with 10 ng/mL trypsin solution (Promega) in 25 mM ammonium bicarbonate, and the tubes were incubated for 12 h at 37 °C. To extract the peptides, the gel pieces were incubated twice with 50 μL of 60% *v*/*v* ACN and 1% *v*/*v* formic acid (FA) and once with 50 μL of ACN. The pellets containing the peptides were resuspended in 0.1% *v*/*v* FA for MS analysis.

### 2.5. Protein Sample Preparation for Gel-Free Methods

Two sets of protein samples were prepared, one for Isobaric Tagging for Relative and Absolute Quantification (iTRAQ, Applied Biosystems, Streetsville, ON, Canada) and the other for label-free protein quantification using mass spectrometry. For iTRAQ analysis, samples containing 50 μg of proteins were processed using the iTRAQ Kit (GE Healthcare, São Paulo, Brazil) according to the manufacturer’s instructions. Proteins were desalted using 1 CC sep pak cartridges (Waters, Milford MA, USA), precipitated overnight using 100% acetone, dissolved in denaturing buffer containing 5 μmol DTT and 2 μL Iodoacetamide, and incubated at 60 °C for 1 h after vortexing. After the addition of 1 μL of cysteine blocking solution (MMTS), the protein solution was further incubated at room temperature for 10 min. Protein samples were digested at 37 °C overnight with trypsin (Promega, Madison, WI, USA), as described by [25]. The digestion was stopped with formic acid (2.5% final concentration), and samples were centrifuged at 20,000 *g* for 20 min. iTRAQ tags were added in a pairwise arrangement, as detailed in Table S1, to minimize labeling artifacts. Samples for each biological replicate were pooled and incubated at room temperature for 1 h prior to mass spectrometry analyses. For label-free quantitative analysis, samples were desalted and digested, as previously described, without the addition of any quantitative tag.

### 2.6. Mass Spectrometry: iTRAQ, Label-Free, and MALDI MS/MS

An aliquot containing 5 µg of peptides (4.5 μL) of either iTRAQ or label-free samples was run through a C18 1.7 μm BEH 130 (100 μm × 100 mm) RP-UPLC (nanoAcquity UPLC, Waters) analytical column coupled to a nano-electrospray tandem Q-Tof PREMIER API mass spectrometer (Waters, Milford, MA, USA), at a flow rate of 600 nL/min. The gradient was 2–90% acetonitrile in 0.1% formic acid for 60 min. The instrument was operated in MS positive mode, data continuum acquisition from *m*/*z* 100–2000 Da, at a scan rate of 1 s^−1^, and an interscan delay of 0.1 s.

Tandem mass spectra were extracted and charge state deconvoluted by Mascot Distiller version 2.4.3 (Matrix Science, London, UK). Deisotoping was not performed. All MS/MS spectra were analyzed using Mascot (version 2.3.02) and X! Tandem (The GPM, thegpm.org; version Cyclone 2010.12.01.1). The Mascot was set up to search the NCBInr green plants (07/2012; 2,229,089 entries), assuming trypsin digestion. X! Tandem was set up to search a subset of the same database, also assuming trypsin differential digestion. Mascot and X! Tandem were searched with a fragment ion mass and parent ion tolerances of 0.100 Da. Carbamidomethylation of cysteine was specified in Mascot and X! Tandem as a fixed modification. Deamidation of asparagine and glutamine and oxidation of methionine were specified as variable modifications. For the iTRAQ samples, iTRAQ-K and iTRAQ-N were set as fixed modifications. iTRAQ-Y and oxidation of methionine were set as variable modifications.

Scaffold (version 4.0.6.1, Proteome Software Inc., Portland, OR, USA) was used to validate mass spectra-based peptide and protein identifications. Peptide identifications were accepted if they could be established with more than 99% probability to achieve a false discovery rate (FDR) <1.0%. Peptide probabilities from X! Tandem were assigned by the peptide prophet algorithm with scaffold delta-mass correction. Protein identifications were accepted if they could be established with more than 95% probability to achieve an FDR <5.0%. Protein probabilities were assigned by the protein prophet algorithm [26]. Proteins that contained similar peptides and could not be differentiated based on mass spectra analyses alone were grouped to satisfy the principles of parsimony. Proteins were annotated using the GO terminology from NCBI using the Blast2Go software [27].

Samples from the 2D gels were analyzed as follows: One 1 uL of sample and 1 uL of alfa-ciano-hydroxycinnamic acid were applied at the MALDI plate. An ABSciex Maldi Tof/Tof 5800 was operated at the full scan, positive mode at 400 Hz for MS and 1000 Hz for MS/MS ions. Ionization source voltage was 4.2 kV in MS mode and 5.4 kV in MS/MS mode. The 1000 shots per spectrum were applied in MS mode and 2500 in MS/MS mode. Mass spectra were processed using protein pilot and Mascot Server; Mascot was set up against either NCBInr green plants, with the same settings described above.

### 2.7. RNA Isolation, Sequencing, and Analyses

Total RNA from root samples was extracted using the TRIzol reagent (Invitrogen, Carlsbad, CA, USA), according to [28]. RNA quantity and integrity were determined using Agilent Bioanalyzer 2100 (Agilent; Palo Alto, CA, USA), according to the manufacturer’s instructions. Only samples with an RNA integrity score >6.6 were used for cDNA synthesis.

cDNA libraries were constructed using 2 µg of total RNA and following the TruSeq RNA Sample preparation v.2 Kit instructions (Low throughput protocol, Illumina, Inc., CA, USA). The quality of the isolated RNA was further determined using a Nanodrop 1000 spectrophotometer (Thermo Scientific, USA) and a Bioanalyzer 2100 (Agilent, Palo Alto, CA, USA). Only samples with an OD 260 to 280 nm ratio between 1.8 and 2.1 and a 28 S/18 S ratio within 1.5–2 were further processed. The TruSeq RNA Sample Prep Kit v2 (Illumina, Inc., CA, USA) was used in the subsequent steps. Briefly, total RNA samples were polyA-enriched, reverse-transcribed, and double-stranded cDNA synthesized. TruSeq adapters were attached to double-stranded cDNA. Enrichment in fragments containing TruSeq adapters on both ends was performed using PCR. The quantity and quality of the enriched libraries were validated using Nanodrop 1000 and Bioanalyzer 2100, respectively. The libraries were normalized to 10 nM of amplicons in 10 mM Tris-HCl, pH 8.5 containing 0.1% Tween 20.

Bar-coded amplicons (library) were spread over one Illumina HiScan 1000 lane. The TruSeq PE Cluster Kit v3-cBot-HS (Illumina, Inc., California, USA) was used for cluster generation using 2 pM of pooled normalized libraries on the cBOT. Sequencing was performed on the Illumina HiScan 1000 to yield paired-end reads of 2 × 101 bases using the TruSeq SBS Kit v3-HS (Illumina, Inc., CA, USA). Reads were quality-checked with Fastqc (http://www.bioinformatics.babraham.ac.uk/projects/fastqc/), which computes various quality metrics for the raw reads.

The CLCBio 6.5 software (Qiagen, Hilden, Germany) was used for *de novo* assembly, Blast searches, annotation, and mapping of the reads. Paired-ended reads (2 × 101 bases) were merged, according to CLCBio default parameters, to produce reads of an average length of 150 bp. Trimming was performed in order to remove ambiguous nucleotides (maximum of 2 nucleotides allowed), terminal nucleotides (1 from the 5′ end and 1 from the 3′ end), adapter sequences, reads with less than 15 and more than 1000 nucleotides, and low-quality reads (limit = 0.02). Contig consensus sequences were compared to the Swissprot plant protein database (http://www.uniprot.org, 11/2013) with an expect level = 10, using BLASTx (NCBI, Bethesda, MD, USA). Subsequently, we produced RNAseq mappings for all reads using the following parameters: minimum read length fraction of 0.9, the minimum similarity of 0.95, allow up to 10 unspecific matches, and select RPKM (Reads Per Kilobase Million) as expression value. Contigs were categorized functionally using Blast2GO PRO [27,29], Mercator [30], and Mapman [31]. To plot the transcriptomic data onto diagrams of metabolic pathways or other processes, the MapMan tool was used [31]. Principal Component Analysis (PCA) was performed with the function prcomp () in R using the gene expressions for all the transcripts with a value different from zero.

To remove transcripts that could be originated from fungus, a Blastn alignment of the contigs was performed against the NCBI database, and those which had more than 90% of similarity to a fungal protein were removed from the analysis.

## 3. Results

A combination of proteomic and transcriptomic analyses was used to compare pooled samples of mycorrhizal (Myc) and non-mycorrhizal (Non-Myc) root segments of the terrestrial orchid *Oeceoclades maculata.* Since the *O. maculata* genome has not been sequenced, protein and gene functions were inferred by homology with sequenced and annotated genomes from other plant species. The dominant fungal symbiont in mycorrhizal roots was also identified by DNA extraction and amplification of the fungal barcoding ITS region.

### 3.1. Fungal Identification

Sequencing of the cloned ITS amplicons indicated the presence of 12 fungal phylotypes in the *O. maculata* Myc roots. Phylotype 1 was the most abundant, comprising approximately 85% of the ITS sequences. BLAST searches showed high similarity to ITS sequences of *Psathyrella candolleana* (Basidiomycota, Accession EU520251, 99% identity, *E*-value 0). Additional phylotypes were represented by single sequences. However, sequence polymorphism was mostly restricted to a single nucleotide substitution, and all phylotypes clustered in the same clade when a phylogenetic NJ tree was constructed with some reference sequences (Figure S1). Sequences from other fungi in the *Agaricomycetes,* known to form OM, clustered, well separate from the *O. maculata* symbiont (Figure S1).

### 3.2. 2D-DIGE Analyses

Using 2D-DIGE, a total of 749 protein spots were detected in Myc and Non-Myc roots (Figure S2). Image analysis and statistical comparisons identified 60 differentially accumulated protein spots (fold change >1.5 or <−1.5; *p* < 0.05) in Myc and Non-Myc root samples. These protein spots were excised from the gels and analyzed by Maldi Q-ToF MS/MS. Out of the 15 proteins identified in Myc roots, 12 were plant proteins, of which six were up-regulated, and six down-regulated (Table S2). Among the most up-regulated proteins in Myc roots were a chaperonin and a heat shock protein, both involved in protein folding, a quinone-oxidoreductase, and an ATP-Binding Cassette (ABC) transporter. Three of the proteins down-regulated in Myc roots (i.e., malate dehydrogenase, glyceraldehyde-3P-dehydrogenase, and enolase) were involved in cell respiration (Table S2).

### 3.3. Label-Free LC-MS/MS and iTRAQ Analyses

Using a label-free quantification approach, we could identify a total of 188 proteins with differential accumulation in Myc and Non-Myc *O. maculata* roots. Only proteins detected in at least two out of three biological replicates were considered as being differentially accumulated, and a total of 88 proteins remained after filtering (Table S3). Among them, 33 proteins were detected exclusively in Myc roots, and 15 showed higher accumulation in Myc roots. Highly represented were hydrolytic enzymes active on protein substrates (e.g., cucumisin, a subtilisin-like serine protease, cysteine, and aspartic proteinases) as well as carbohydrates (e.g., alpha-glucosidase, alpha-mannosidase, arabinosidase, 1,3-beta-glucosidase). By contrast, 39 proteins were exclusively detected in Non-Myc roots, and eight showed higher accumulation in Non-Myc roots (Table S3). Among them were the same enzymes involved in cell respiration (either glycolysis or the Krebs cycle) that were already identified by the 2D DIGE analysis, thus confirming the results. Interestingly, a fructokinase was exclusively found in Non-Myc roots. This enzyme has been proposed to regulate starch synthesis in sink tissues [32]. Using the iTRAQ approach, 168 proteins were identified, of which 86 showed differential accumulation (fold change >1.5 or <−1.5). However, only 10 proteins showed a statistically significant (Mann–Whitney test, *p* < 0.05) differential accumulation among replicates (Table S4).

**Figure 2:**
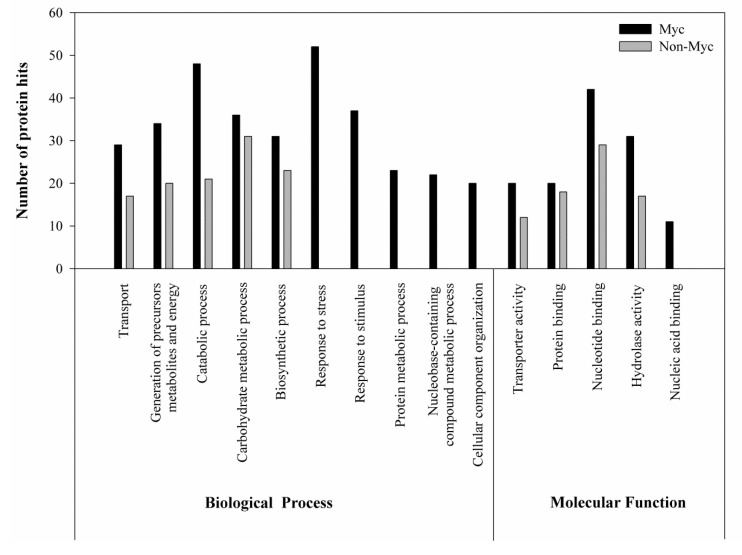
Gene ontology (GO) assignments of biological processes and molecular function for proteins homologous to plant proteins derived from label-free LC-MS/MS proteomics of mycorrhizal (Myc) and non-mycorrhizal (Non-Myc) roots of *Oeceoclades maculata.*

Proteins identified in the label-free LC-MS/MS were categorized by gene ontology (GO) annotation and the Blast2Go algorithm (Figure 2). Proteins from Myc roots could be classified into 10 categories of biological functions, whereas only five categories were represented in Non-Myc roots. The most abundant categories in Myc roots were “response to stress” (16% of proteins) and “catabolic process” (15% of proteins), whereas the most abundant category (28% of proteins) in Non-Myc roots was “carbohydrate metabolic process”.

### 3.4. Transcriptomic Analyses

**Figure 3:**
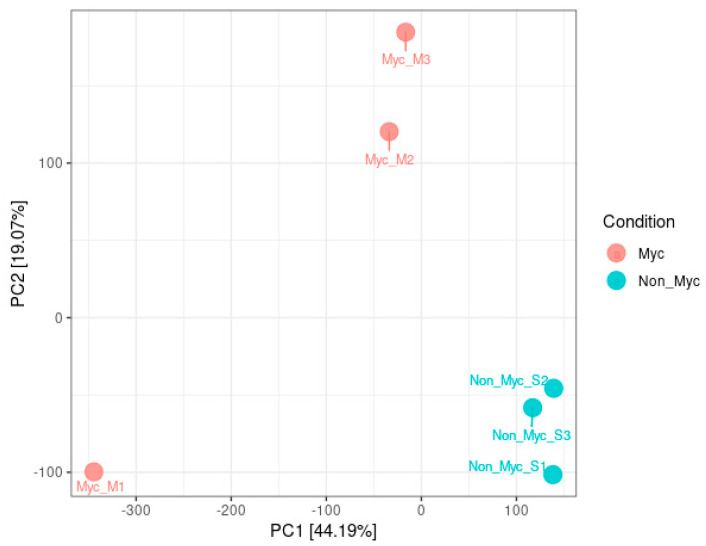
Principal Component Analysis (PCA) of the complete transcriptomes of the three biological replicates of mycorrhizal (Myc) and non-mycorrhizal (Non-Myc) root samples of *Oeceoclades maculata.*

cDNA sequencing produced approximately 83 million reads with a mean length of 101 bases and a mean quality score of 38 (Phred Score). Information on sequence merging and trimming is shown in Table S5. After the removal of reads containing low-quality and/or ambiguous bases, reads were clustered in high-quality contigs by *de novo* assembly (Table S6). Out of the 78,137 contigs, 311 had a similarity above 90% to fungal sequences in the NCBI database and were removed from the database. An average of 89.6% of the total reads was successfully mapped back to the contigs (89.1% of single reads and 90.1% of paired reads). When the plant transcriptomic profiles of Myc and Non-Myc root samples were compared by PCA, the Non-Myc root samples clustered together, well separate from the Myc samples, where a higher variability was observed (Figure 3). Data were normalized, and a *t*-test was applied to statistically evaluate differential accumulation between Myc and Non-Myc roots of *O. maculata*. Contigs with fold-change >1.5 or <−1.5 (*p* < 0.05) were considered to be differentially accumulated. Of the plant contigs generated by *de novo* assembly, 7.14% (5854) were up-regulated, and 4.30% (3514) were down-regulated in mycorrhizal roots, although Blast2Go annotation could only identify 1359 up-regulated and 751 down-regulated contigs.

### 3.5. Regulated Plant Transcripts in Mycorrhizal O. maculata Roots

**Figure 4:**
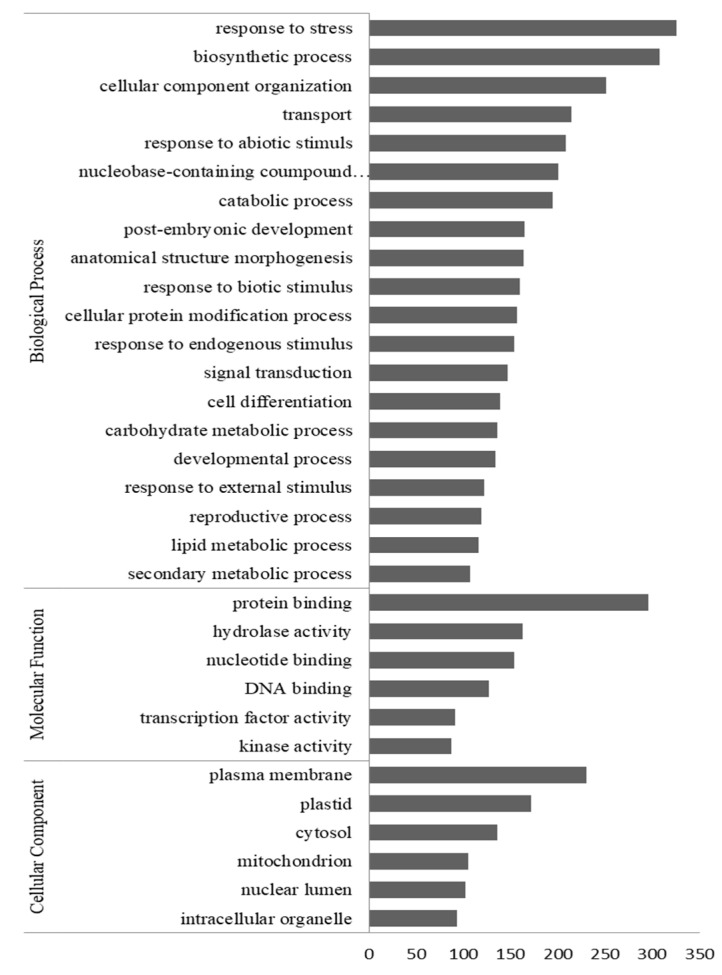
Gene ontology (GO) categorization of transcripts up-regulated (fold change >1.5; *p* < 0.05) in mycorrhizal roots of *Oeceoclades maculata.* Bars indicate the number of reads. X-axis, the number of reads.

Up-regulated plant transcripts in Myc roots of *O. maculata* were assigned to 20 different “biological processes”, six “cellular components”, and six “molecular functions” categories, according to GO searches (Figure 4). Among the biological processes, “response to stress” was the most represented category, followed by the “biosynthetic process”, “cellular component organization”, and ”transport”. Among the molecular functions, most transcripts were associated with “protein binding”, “hydrolase activity”, and “nucleotide binding”. Concerning the sub-cellular localization, most up-regulated transcripts in mycorrhizal roots encoded putative plasma membrane proteins. Up- and down-regulated plant transcripts successfully identified by BlastX in the Swissprot plant protein database and annotated by Blast2Go are listed in Table S7.

**Figure 5:**
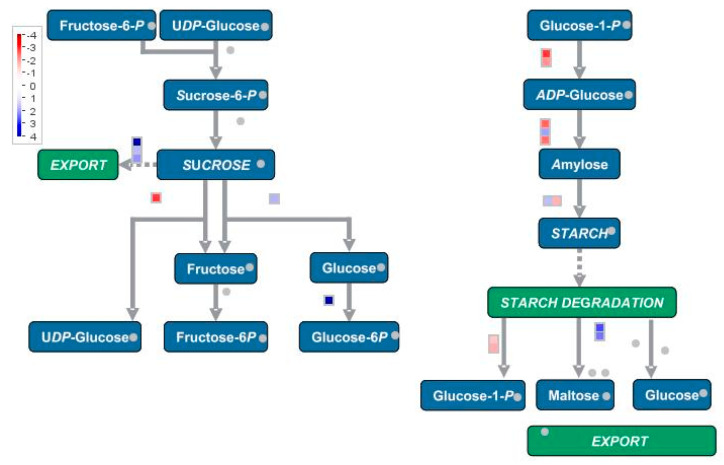
Pathway level display of genes involved in sucrose and starch metabolism, produced with MapMan [31]. Changes of transcripts levels in Myc and Non-Myc *Oeceoclades maculata* roots are indicated in the squares. Blue squares represent genes up-regulated, whereas red squares represent genes down-regulated in Myc roots, as compared with Non-Myc roots. Grey dots represent genes that were not mapped in the current dataset. A reference scale is on the left.

These regulated transcripts were also classified using KEGG (Kyoto Enciclopedia of Genes and Genomes, Kanehisa Laboratories, Kyoto University, Kyoto, Japan) annotations, which provide an alternative functional annotation of genes according to their associated biochemical pathways, based on sequence similarity searches against the KEGG database (http://www.genome.jp/kegg/pathway.html). “Starch and sucrose metabolism” was the most represented pathway, with 31 transcripts representing 20 enzymes in the KEGG map. Enzymes encoded by up-regulated transcripts in Myc roots are illustrated in Figure S3. To illustrate the regulation of starch and sucrose metabolism in *O. maculata* roots, a MapMan visualization is also shown in Figure 5. Genes identified as being involved in starch biosynthesis were mainly up-regulated in Non-Myc roots, whereas genes involved in starch degradation and maltose release, as well as sucrose export, were mostly up-regulated in Myc roots (Table S7 and Figure 5). Well-represented up-regulated transcripts in Myc roots were also involved in other metabolic pathways, such as “purine metabolism”, with 26 detected transcripts representing 15 enzymes in the KEGG map, and “glyoxylate and dicarboxylate metabolism”, with 11 detected transcripts representing seven enzymes.

Individual transcripts in Table S7 provided additional information on metabolism and nutrient exchanges in mycorrhizal *O. maculata* roots. Some of the transcripts up-regulated in Myc roots coded for proteins involved in the transport of mineral nutrients (Table S7). In particular, two inorganic phosphate transporters (IDs Q8H6G8 and Q8H6H2) likely located on the plasma membrane were up-regulated in Myc roots (fold change FC) = 10.7 and 6.8, respectively). An ammonium transporter (ID Q6K9G1, FC = 9.5), two amino acid permeases (ID Q42400, FC = 51.7 and IDQ38967, FC = 1.7), a cationic amino acid transporter (ID Q84MA5, FC = 6.7), and a lysine histidine transporter (ID Q9FKS8, FC = 4.5), all likely located on the plasma membrane, were also up-regulated in Myc roots (Table S7).

The most abundant transcripts up-regulated in Myc roots (Table S7) coded for a protein similar to syntaxin-24 (Syp24, ID Q9C615, FC = 2.9), and two other less abundant syntaxins (Syp121 and Syp131) were also significantly up-regulated (ID Q9ZSD4, FC = 3.2 and ID Q9SRV7, FC = 1.7). Syntaxins are members of the SNARE superfamily proteins [33].

**Figure 6:**
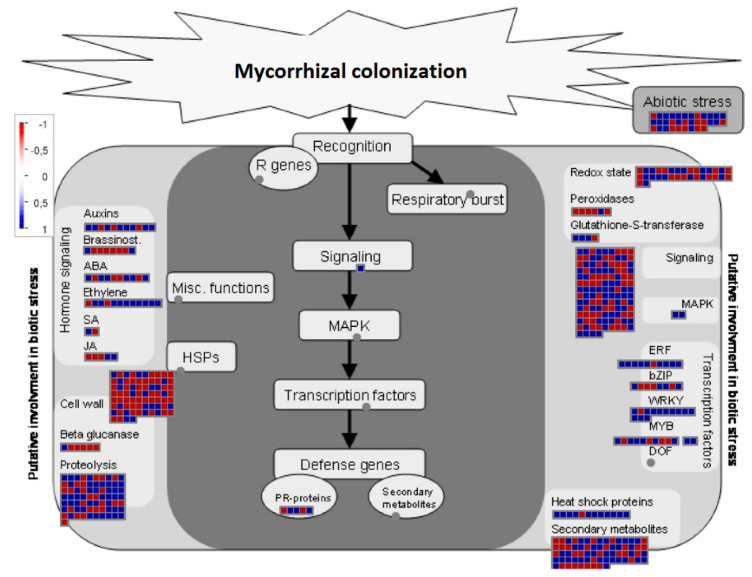
Biotic stress responses are illustrated by MapMan [29] and based on the list of differentially regulated genes in *Oeceoclades maculata* roots (Table S7). Blue squares represent genes up-regulated, whereas red squares represent genes down-regulated in mycorrhizal roots, as compared with non-mycorrhizal roots. A scale is on the left.

In addition to changes in cell metabolism and nutrient exchanges, the establishment and functioning of the mycorrhizal symbiosis likely involve a strict control of the fungus by the host plant. We provided, therefore, as a MapMan heatmap, an overall graphical representation of regulated genes potentially involved in biotic and abiotic stress responses in Myc and Non-Myc *O. maculata* roots (Figure 6). This MapMan visualization of transcriptional profiles was developed for Arabidopsis [31] but has already been used to illustrate plant-microbe interactions in several other plant species [34].

Most transcripts coding for proteins acting on the plant cell wall were significantly more expressed in Non-Myc roots than in Myc roots (Figure 6). Among them, a CTL1 chitinase-like protein was strongly down-regulated (ID Q9MA41, FC = −11.6) in Myc roots. A fasciclin-like arabinogalactan protein (ID O22126, FC = −6.6) and a pectin methylesterase inhibitor (ID Q9M3B0, FC = −6.5), also involved in plant cell wall biogenesis or remodeling, were among the most down-regulated plant transcripts in Myc roots (Table S7).

Among the plant transcripts significantly down-regulated in Myc roots (Table S7), the most strongly down-regulated (ID B2KNH9, FC = −37.9), as well as the most abundant in Non-Myc roots, coded for a mannose-specific lectin. Mannose-specific lectins have been shown to be involved in antifungal responses in the orchid *Gastrodia elata* [35]. Conversely, one of the most highly up-regulated (ID Q6K5Q0, FC = 71.6) transcripts in Myc roots coded for a germin-like protein, also implicated in antioxidative responses and plant response to biotic and abiotic stress [36].

Several transcription factors, such as ethylene-responsive transcription factors, MYB-related, WRKY homologs, or containing MADS-box (MCM1 AGAMOUS DEFICIENS SRF), were found to be regulated at the transcriptional level, most of them being up-regulated in Myc roots (Table S7). Many of these transcription factors are involved in defense response to biotic or abiotic stress and are considered in Figure 6.

Hormone signaling is an important component of plant response to stress. Whereas transcripts encoding ethylene-responsive transcription factors (e.g., IDs Q9C9I2, FC = 8.35; Q6J9S1, FC = 3.82; Q1PFE1, FC = 3.64) were mostly up-regulated in Myc roots (Table S7 and Figure 6), transcripts encoding an allene oxide synthase (ID P48417, FC = −4.33) and a 9-lipoxygenase (ID Q43191, FC = −5.71), both involved in the biosynthesis of the oxylipin jasmonate (JA), were strongly down-regulated in mycorrhizal roots. Interestingly, a jasmonate zim-domain (JAZ) protein, a repressor of JA responsive genes, was up-regulated (ID Q9S7M2, FC = 4.04) in Myc roots (Table S7).

## 4. Discussion

Molecular aspects of orchid mycorrhizal interactions in adult roots have received very little attention [16,37], and most information on this symbiosis derives from in vitro experiments with germinated seeds and protocorms [10,11,13,14,38]. However, the relationship of mycoheterotrophic orchid protocorms with the mycorrhizal fungal partner is expected to be quite different from that of adult plants, especially in species developing photosynthetic leaves, such as *O. maculata*. Therefore, we investigated changes in protein and gene expression occurring in field-collected root samples of adult *O. maculata* plants. The main fungal symbiont in *O. maculata* mycorrhizal roots was identified as *Psathyrella candolleana* (Basidiomycota). *Psathyrella* species are common fungal saprotrophs found in soil [39], and they have been already reported in the mycoheterotrophic orchids *Epipogium roseum* [40] and *Eulophia zollingeri* [41]. *Psathyrella candolleana* has been recently described as a mycorrhizal symbiont of *O. maculata* in Puerto Rico [42], and we confirmed the occurrence of the same species in Brazil. Although Bayman et al. [42] could also identify additional orchid mycorrhizal fungi (e.g., *Ceratobasidium* and *Tulasnella*), *O. maculata* seeds germinated in vitro only in the presence of *P. candolleana*. We could not identify other mycorrhizal symbionts, but it should be noted that the ITS primers we used do not amplify certain groups of *Tulasnella* because of rapid sequence evolution [43]. As we did not isolate *P. candolleana* in pure culture, we could not compare fungal gene expression under symbiotic and asymbiotic conditions, and we, therefore, focused on the plant responses to mycorrhization.

Our transcriptomic and proteomic data allowed the identification of important changes in root metabolism due to the OM symbiosis. The different proteomic approaches allowed us to identify about 300 plant proteins in total, whereas the transcriptome allowed the identification and quantification of more than 70,000 contigs. Despite the substantial differences in the coverage of the two approaches, due to the constraints of the proteomic techniques, the results were coherent, and both approaches added substantial biological information to this study. The proteome was useful to highlight proteins that accumulated in high amounts in the roots, whereas the RNAseq added depth and coverage to the analysis, confirming most of the proteomic data and complementing plant pathways that were pointed out but not fully represented in the proteome.

### 4.1. Changes in the Energetic Metabolism in Orchid Mycorrhizal Roots

Both proteomic and transcriptomic results indicated that starch degradation and sugar transport were favored in *O. maculata* mycorrhizal roots, whereas starch biosynthesis was favored in non-mycorrhizal roots. This result provided a molecular explanation to the presence of large amounts of starch granules in non-mycorrhizal roots of *O. maculata* and raised some intriguing questions on the origin of this storage polysaccharide. The role of mycorrhizal fungi in providing organic carbon to the host plant during seed germination and protocorm development is probably one of the key features and first observed phenomena in OM (see in [44]), but [7,8] demonstrated that the net flow of carbon was reversed in the adult photosynthetic orchid *Goodyera repens*. As in arbuscular mycorrhiza, starch biosynthesis in the non-mycorrhizal roots of *O. maculata* may, therefore, use photosynthesis-derived sugars to store carbon that may be later transferred to the mycorrhizal fungus. Indeed, starch accumulation in *Lotus japonicus* roots was induced when fungal colonization was prevented, whereas mycorrhizal root colonization led to a decrease in starch accumulation [45]. We could not exclude that at least some of the carbon accumulated as starch in the younger non-mycorrhizal roots of *O. maculata* might derive indirectly from the mycorrhizal fungus. Although *O. maculata* is green and photosynthetic, partial mycoheterotrophy has been suggested for this species, which is common in dense shade and associates with a dominant fungal symbiont known to colonize only mycoheterotrophic orchids [42]. Thus, although its trophic strategy remains to be assessed, *O. maculata* may be able to exploit the mycorrhizal symbiont for organic carbon, which could be exported from mycorrhizal roots and redistributed to other plant tissues and to younger non-mycorrhizal roots. Up-regulated plant transcripts in mycorrhizal roots include sugar (hexose and sucrose) transporters and a bidirectional sugar transporter of the SWEET2a family. SWEETs mediate both low-affinity uptake and efflux of sugars across the plasma membrane. They have been reported in both orchid [38] and arbuscular [46] mycorrhiza, but although they could play an important role in regulating the transfer of carbon at the plant-fungus interface, their role in symbiosis is far from clear.

### 4.2. Nutrients Transport in Orchid Mycorrhizal Roots

In addition to carbon, OM fungi are known to provide their host plant with mineral nutrients, and the higher frequency of proteins involved in “transport” in the *O. maculata* Myc root proteome and transcriptome suggests an enhanced solute exchange at the plant-fungus interface. Two transcripts up-regulated in mycorrhizal roots code for phosphate transporters, in line with the evidence of mycorrhizal acquisition of inorganic phosphorus in adult plants of the terrestrial orchid *Goodyera repens* [47]. An inorganic transporter was also reported to be up-regulated in mycorrhizal roots of the epiphytic orchid *Cymbidium hybridum* [37], although this transporter could be also induced by the non-mycorrhizal fungus *Umbelopsis nana*.

Nitrogen concentrations in the shoots of terrestrial orchids can be surprisingly high, especially in achlorophyllous species, which would be expected to have a lower nitrogen demand [48]. Cameron et al. [7] were the first to demonstrate that nitrogen, when supplied as glycine, was transferred from the fungal mycelium to roots, rhizomes, and shoots of *G. repens.* An ammonium transporter and several amino acid transporters were significantly up-regulated in mycorrhizal roots of *O. maculata*, suggesting that OM increases nitrogen uptake and that amino acids may be an important source. Fochi et al. [14] recently reported a strong induction of amino acid transporters in mycorrhizal protocorms of *Serapias vomeracea* colonized by *Tulasnella calospora*. In particular, the most up-regulated transporter was a lysine histidine transporter, mostly expressed in mycorrhizal cells containing viable fungal hyphae [49]. A lysine histidine transporter was also up-regulated in mycorrhizal roots of *O. maculata*. Overall, our results indicate that amino acids provided by the fungal partner might remain an important source of nitrogen (and perhaps carbon) also for adult orchids and that some amino acids might be preferentially transferred, irrespective of the taxonomic position of the symbiotic fungus and the plant developmental stage.

### 4.3. Redox Homeostasis and Plant Defense Responses

Cellular redox homeostasis is a central modulator in plant stress responses to biotic and abiotic factors [50]. The generation of reactive oxygen species (ROS) and their homeostatic regulation by antioxidant enzymes have been frequently observed in plant-microbe interactions [51,52,53], but there was no clear evidence of induction of genes and proteins involved in redox homeostasis in *O. maculata* mycorrhizal roots. Although higher levels of some enzymes known to be involved in redox homeostasis regulation [54,55,56] were detected in mycorrhizal roots by proteomics, such as quinone-oxidoreductase, peroxidase, glutathione reductase, and ascorbate peroxidase, proteins and transcripts similarly involved in redox homeostasis were also up-regulated in non-mycorrhizal roots. Thus, mycorrhizal colonization did not seem to strongly influence redox homeostasis in the host plant. Somewhat different results were obtained by Zhao et al. [37] in *C. hybridum* roots, where several transcripts encoding stress-responsive proteins or proteins involved in redox homeostasis were induced in mycorrhizal roots. However, unlike the experiment in *C. hybridum*, where mycorrhizal individuals were compared with non-mycorrhizal ones [37], mycorrhizal and non-mycorrhizal *O. maculata* root samples were collected from the same plant individuals. Therefore, we could not exclude that a systemic response to fungal colonization could have been overlooked in our experiment.

Plant defense responses to colonization by pathogenic fungi usually involve the induction of enzymes that can degrade the fungal cell wall, as well as pathogenesis-related (PR) proteins and proteins involved in plant cell wall modification [57,58]. Although two PR proteins were found by proteomics to be more expressed in mycorrhizal roots of *O. maculata*, transcriptomic data indicated that plant defense responses were lower in mycorrhizal roots than in non-mycorrhizal roots. Plant lectins are carbohydrate-binding proteins that have been also demonstrated to play a role in plant defense against invading microbes [59]. In particular, mannose-binding lectins have been identified in several orchid species, and their antifungal activity has been demonstrated *in vitro*. For example, mannose-binding proteins found in *Gastrodia elata* and *Epipactis helleborine* displays in vitro antifungal activity against *Rhizoctonia solani* and *Phytophthora nicotianae* [35], and the agglutinin produced by *Dendrobium findleyanum* inhibits the growth of *Alternaria alternata* [60]. Transcripts coding for a mannose-binding lectin were the most strongly down-regulated transcripts in mycorrhizal roots, indicating that this line of defense is not activated in colonized roots. However, an alternative explanation is also possible, and the high levels of transcripts encoding proteins with antifungal properties in non-mycorrhizal organs may reflect a mechanism to locally prevent fungal growth in storage organs. Orchids produce a plethora of secondary metabolites [61], and a similar role in the limitation of mycorrhizal fungal invasion in storage organs has been suggested for antifungal secondary metabolites produced by several orchid species [62].

Chitinases can degrade the fungal cell wall and are part of a general line of defense strongly induced as a response to colonization by pathogenic fungi. The only regulated chitinase was a chitinase-like protein 1 (CTL1) identified in the transcriptome and strongly down-regulated in mycorrhizal roots. BlastX searches confirmed the identification, the closest match being a *Phalaenopsis equestris* CTL1 (58% query coverage; *E*-value = 0; 94.12% identity). However, its role remains unclear because molecular and biochemical analyses showed that CTL1 had no apparent chitinase enzymatic activity [63]. In Arabidopsis, CTL1 is a glucan-interacting protein important for cellulose assembly and interaction with hemicelluloses [64]. The role during mycorrhizal interaction in *O. maculata* remains to be established.

### 4.4. Hormone Regulation

Plant responses to fungal symbionts involve changes in phytohormone balance. Among the plant hormones, jasmonic acid (JA), ethylene (ET), and abscisic acid (ABA) are known to fine-tune plant defense responses during interactions with mycorrhizal fungi [65,66]. Jasmonate is an important regulator of plant responses to biotic and abiotic stresses and is considered essential for defense responses against various pathogens and herbivorous insects [67]. Proteomic data showed that two allene oxide synthases, which catalyze the first step in jasmonic acid biosynthesis, and a lipoxygenase, which catalyzes the biosynthesis of oxylipins, precursors of several metabolites, including JA, were both identified exclusively in non-mycorrhizal roots of *O. maculata*. Endogenous levels of JA below a threshold concentration switch off the expression of JA-responsive genes through active repression of specific transcription factors by jasmonate zim-domain (JAZ) proteins [68]. Interestingly, the proteomic data identified a JAZ protein only in *O. maculata* mycorrhizal roots. Corroborating the proteomic data, transcripts encoding a JAZ protein were found to be up-regulated in mycorrhizal roots, whereas transcripts related to an allene oxide synthase and 9-lipoxygenase were down-regulated in mycorrhizal roots. Gutjahr et al. [69] showed that JA was not required for arbuscular mycorrhizal (AM) colonization, and high levels of JA suppressed AM development likely due to the induction of plant defense. Our results suggested a similar scenario in orchid mycorrhiza, where the suppression of JA biosynthesis might be important to allow fungal colonization.

Ethylene is another key phytohormone involved in the regulation of both plant defense and mutualistic plant-microbe interactions [70,71]. In our transcriptome, 15 ethylene-responsive transcription factors and three ethylene-induced calmodulin genes were up-regulated in *O. maculata* mycorrhizal roots, thus suggesting that ethylene plays a role in orchid mycorrhiza. Similar results were also reported by Zhao et al. [37], who identified transcripts related to ET metabolism and signaling in mycorrhizal roots of *C. hybridum*.

## 5. Conclusions

We identified transcriptomic and proteomic changes in orchid metabolism in response to mycorrhizal colonization in the adult orchid *O. maculata*. Not surprisingly, mycorrhizal colonization was accompanied by significant local changes in the host plant metabolism, especially concerning carbon and nitrogen metabolism. In particular, the up-regulation of some amino acid transporters would suggest the preferential use of organic nitrogen, which might be transferred by the orchid mycorrhizal fungus across the plant-fungus interface not only at the protocorm stage [14], but also in adult orchids.

Plants forming intracellular symbioses share a common genetic toolkit irrespective of the taxonomic position of the symbiotic partner [72]. This finding may explain why, despite the distant taxonomic position of the fungal symbionts, some plant responses were common in AM and OM, especially concerning the modulation of defense responses in mycorrhizal roots. Similar to AM, root colonization by OM fungi was mirrored in fact by a lower expression of plant defenses, which allowed the host cells to accommodate the fungal partner and to develop a functional symbiosis. The down-regulation of transcripts involved in JA transduction pathways in *O. maculata* mycorrhizal roots might lead to the lower antifungal responses observed. The strong antifungal responses and the absence of fungal growth in non-mycorrhizal tissues might be also related to higher JA levels.

In conclusion, *O. maculata* proved to be an interesting model for investigating mycorrhizal interactions in adult orchids, and further studies should address specific roles of the different components of the orchid symbiotic machinery.

## Supplementary Materials

The following are available online at https://www.mdpi.com/2309-608X/6/3/148/s1, Figure S1: Neighbor-joining phylogenetic tree of ITS fungal sequences obtained from mycorrhizal roots of *O. maculata*. Figure S2: 2D-DIGE of proteins isolated from mycorrhizal and non-mycorrhizal roots of *O. maculata*. Figure S3. Mapping on the “starch and sucrose metabolism” KEGG pathway of genes up-regulated in the mycorrhizal roots of *O. maculata,* as compared with Non-Myc roots. Table S1: iTRAQ labeling scheme for forward and reverse tagging of three biological replicates of *Oeceoclades maculata* roots. Table S2: Significant protein hits from differentially accumulated spots of mycorrhizal and non-mycorrhizal roots of *O. maculata* with the 2D-DIGE technique. Table S3: Proteins identification and quantification in mycorrhizal roots of *O. maculata* using the label-free LC-MS/MS approach. Table S4: Relative quantification of proteins with significant differential accumulation in mycorrhizal and non-mycorrhizal roots of *O. maculata* using iTRAQ. Table S5: Statistics of cDNA sequences derived from *O. maculata* roots. Table S6: Length distribution of de novo assembled contigs. Table S7: List of transcripts significantly regulated in Myc vs. Non-Myc roots of *O. maculata* (Excel file).
