# Supplementary material for: Proteomic and Transcriptomic Analyses Indicate Metabolic Changes and Reduced Defense Responses in Mycorrhizal Roots of Oeceoclades maculata (Orchidaceae) Collected in Nature
Source: J Fungi (Basel). 2020 Aug 26;6(3):148. doi: 10.3390/jof6030148 (PMC7558880; doi:10.3390/jof6030148)
Supplement: Supplementary file 1 [file jof-06-00148-s001.zip › Valadares_Supplementary_Files/Valadares_Supplementary_JoF_revised.docx]

Proteomics and transcriptomics indicate metabolic changes and reduced defense responses in mycorrhizal roots of *Oeceoclades maculata* (Orchidaceae) collected in nature

Rafael B. S. Valadares ^1,6,^*, Silvia Perotto ^2,^*, Adriano R. Lucheta ^3^, Eder C. Santos ^4^, Renato M. Oliveira^5,6^ and Marcio R. Lambais ^1^

Supplementary Material

**Figure S1**: Neighbour joining phylogenetic tree of ITS fungal sequences obtained from mycorrhizal roots of *O. maculata*.
**Figure S2**: 2D-DIGE of proteins isolated from mycorrhizal and non-mycorrhizal roots of *O. maculata*.
**Figure S3**. Mapping on the “Starch and Sucrose Metabolism” Kegg Pathway of genes up-regulated in the mycorrhizal roots of *O. maculate,* as compared with Non-Myc roots.

**Table S1**: iTRAQ labeling scheme for forward and reverse tagging of three biological replicates of *Oeceoclades maculata* roots,
**Table S2**: Significant protein hits from differentially accumulated spots of mycorrhizal and non-mycorrhizal roots of *O. maculat*a with the 2D-DIGE technique.
**Table S3**: Proteins identification and quantification in mycorrhizal roots of *O. maculata* using the Label free-LC MS/MS approach
**Table S4**: Relative quantification of proteins with significant differential accumulation in mycorrhizal and non-mycorrhizal roots of *O. maculata* using iTRAQ
**Table S5**: Statistics of cDNA sequences derived from *O. maculata* roots
**Table S6**: Length distribution of *de novo* assembled contigs
**Table S7**: List of transcripts significantly regulated in Myc vs Non-Myc roots of *O. maculata* (Excel file)

Proteome Label-free and Itraq spectrum list (.mgf) and mascot result file (.dat) has been deposited under this code: **MassIVE MSV000085842**

Link for ftp download: <ftp://massive.ucsd.edu/MSV000085842/>

Transcriptomic data have been deposited in the GEO repository (**GSE155822**)

| 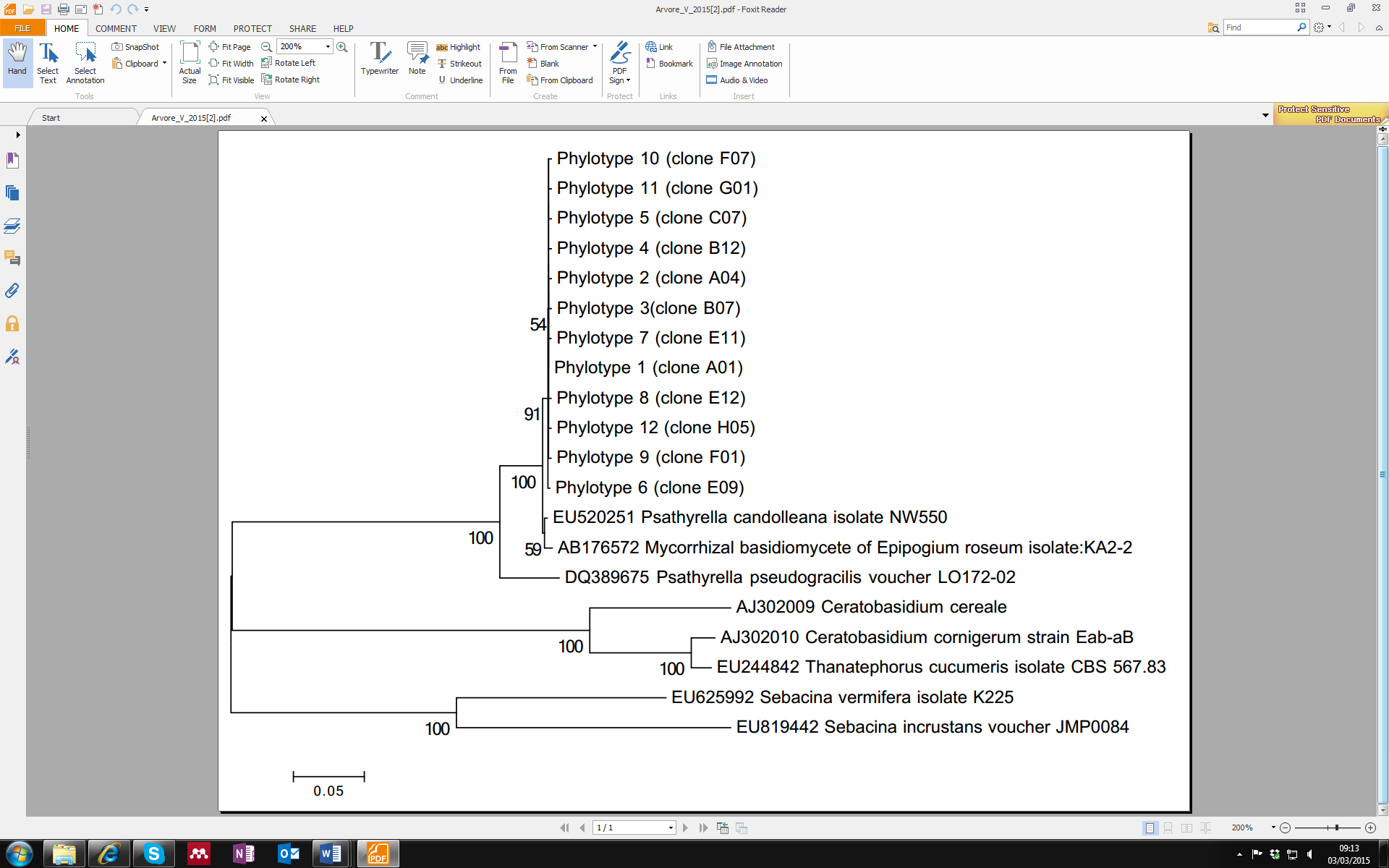 |
| --- |

**Figure S1:** Neighbour joining phylogenetic tree of ITS fungal sequences obtained from mycorrhizal roots of *O. maculata*. ITS sequences of best matches from Blast search against NCBI database [21] as well as of other fungi known to form orchid mycorrhiza were used. The NJ tree was obtained using Kimura-two parameter model followed by bootstrap test (1,000 replicates).

| 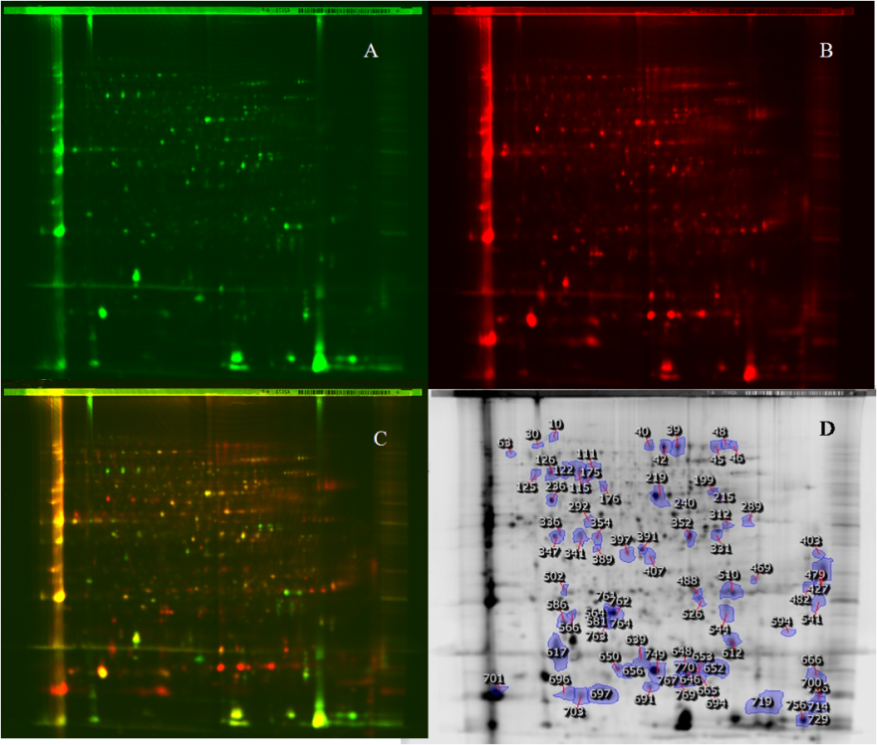 |
| --- |

**Figure S2:** 2D-DIGE of proteins isolated from mycorrhizal and non-mycorrhizal roots of O. maculata. All images refer to the same gel but scanned with different wavelengths. **A**: Proteins from non-mycorrhizal roots stained with Cy3. **B**: Proteins from mycorrhizal roots stained with Cy5. **C**: Overlaid images of **A** and **B**. **D**: Spot picking list

| 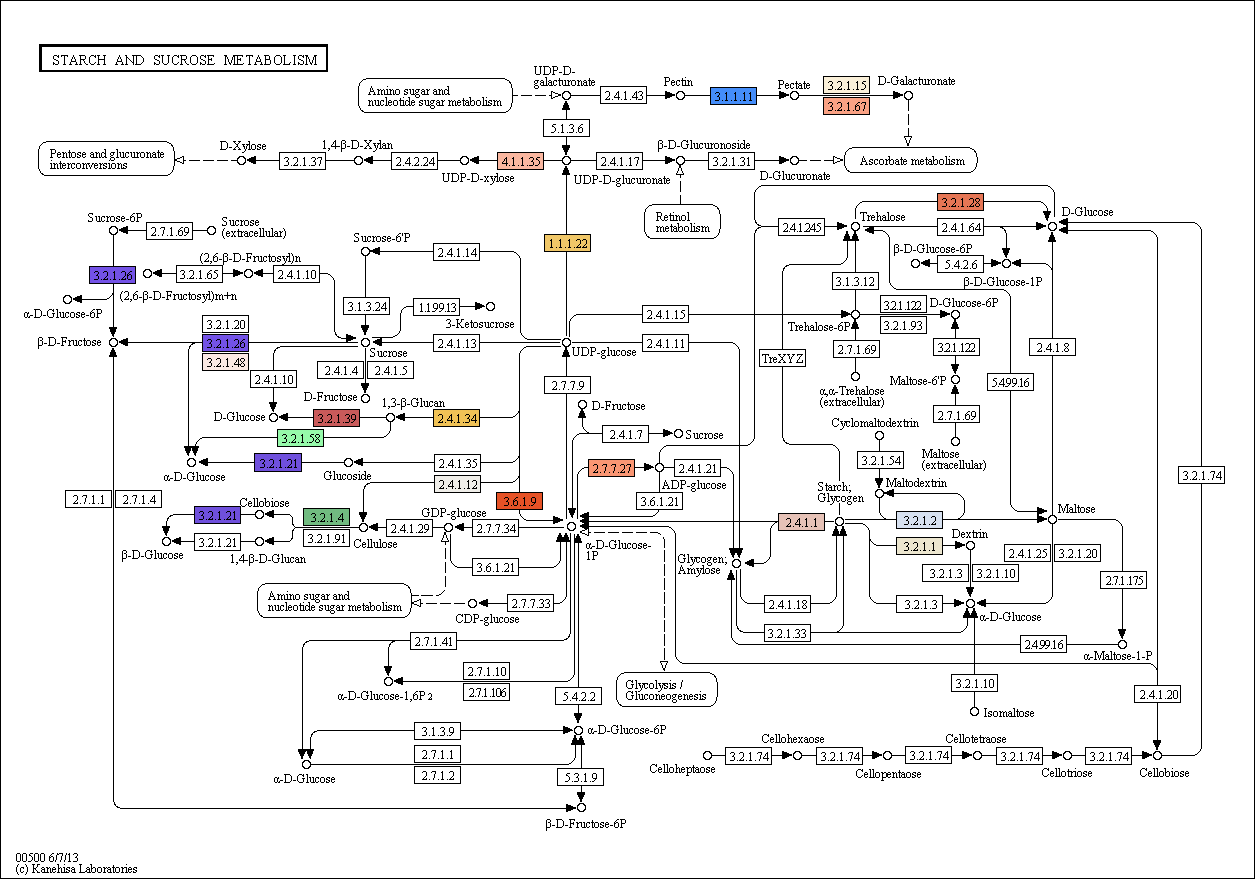 |
| --- |

**Figure S3:** Mapping on the “Starch and Sucrose Metabolism” Kegg Pathway of genes up-regulated in the mycorrhizal roots of *O. Maculata*, as compared with Non-Myc roots. Colored enzyme codes represent *O. maculata* transcripts with fold change >1.5 (*p-value* <0.05).

**Table S1.** iTRAQ labeling scheme for forward and reverse tagging of three biological replicates of *Oeceoclades maculata* roots. 114, 115, 116 and 117 refers to different iTRAQ tags.

|  | **Sample** | **Mycorrhizal** | **Non-mycorrhizal** |
| --- | --- | --- | --- |
| Biological replicate 1 | A | 114 | 115 |
|  | B (reverse) | 115 | 114 |
| Biological replicate 2 | C | 114 | 115 |
|  | D (reverse) | 115 | 114 |
| Biological replicate 3 | E | 116 | 117 |
|  | F (reverse) | 117 | 116 |

**Table S2.** Significant plant protein hits from differentially accumulated spots of mycorrhizal and non-mycorrhizal roots of *O. maculata* (fold change > 1,5; *p* < 0.05), with the 2D-DIGE technique.

| **Spot ID** | **Anova** | **Fold Change^1^** | **Best Protein Accession** | **Best Protein Mass** | **Best Protein Score** | **Best Protein Description** |
| --- | --- | --- | --- | --- | --- | --- |
| 586 | 0.009 | 5.1 | ref\|XP_002277861.1\| | 2630529 | 156 | Chaperonin |
| 762 | 0.003 | 5.2 | gb\|ABD98056.1\| | 2184808 | 90 | Quinone-oxidoreductase QR2 |
| 42 | 0.013 | 5.4 | ref\|XP_002518324.1\| | 7130398 | 87 | Heat shock protein |
| 391 | 0.004 | 4.3 | ref\|XP_001419026.1\| | 2372541 | 77 | ABC family transporter |
| 469 | 0.048 | 3.7 | emb\|CAN60148.1\| | 2643814 | 73 | Dienelactone hydrolase family |
| 199 | 0.019 | 2.9 | F2YP47_VANPL | 5004591 | 77 | Beta-tubulin |
| 594 | 0.029 | -2 | gb\|ABC55649.1\| | 1912855 | 98 | Translationally controlled tumor protein |
| 666 | 0.013 | -1.6 | ref\|XP_002866880.1\| | 2034206 | 90 | Lipid-associated family protein |
| 219 | 0.003 | -1.6 | sp\|P84541.1\|ENO_POPEU | 274941 | 76 | Enolase |
| 336 | 0.015 | -1.8 | MDHC_BETVU | 3581036 | 67 | Malate dehydrogenase |
| 354 | 0.015 | -1.6 | G9FP71_9ASPA | 3134526 | 63 | Glyceraldehyde 3P dehydrogenase |
| 352 | 0.002 | -1.6 | LGUL_BRAOG | 3174010 | 94 | Lactoylglutathione lyase |
|  |  |  |  |  |  |  |

^1^ Fold Change = Mycorrhizal /Non-mycorrhizal normalized peak intensity values.

**Table S3.** Proteins identification and quantification in mycorrhizal roots of *O. maculata* using the Label free-LC MS/MS approach. Non-mycorrhizal roots were taken as reference (Ref) samples. Identification probability are written in terms of percentage in each biological replicate.

| **Protein ID** | **Accession** | **MW** | **FC** | **Myc1** | **Myc2** | **Myc3** | **Ref** | **Ref** | **Ref** |
| --- | --- | --- | --- | --- | --- | --- | --- | --- | --- |
| jasmonate ZIM-domain protein 2 | gi\|196259696 | 32 kDa | **Myc** | 0 | 95% | 98% | 0 | 0 | 0 |
| cucumisin-like | gi\|359486591 | 81 kDa | **Myc** | 100% | 100% | 100% | 0 | 0 | 0 |
| NADH-dependent enoyl-ACP reductase* | gi\|75225229 | 47 kDa | **Myc** | 100% | 100% | 100% | 0 | 0 | 78% |
| cyclophilin | gi\|192910744 | 18 kDa | **Myc** | 100% | 100% | 100% | 0 | 0 | 0 |
| hypothetical protein SORBIDRAFT_01g050550 | gi\|241919896 | 81 kDa | **Myc** | 0 | 95% | 94% | 0 | 0 | 0 |
| cysteine proteinase | gi\|148927394 | 51 kDa | **Myc** | 93% | 100% | 100% | 0 | 0 | 0 |
| alpha-glucosidase | gi\|15239154 | 101 kDa | **Myc** | 100% | 100% | 100% | 0 | 0 | 0 |
| NADH dehyd. [ubiquinone] Fe-Su protein 7* | gi\|259491355 | 23 kDa | **Myc** | 99% | 99% | 99% | 0 | 0 | 0 |
| cell division cycle protein 27 homolog B | gi\|75330104 | 81 kDa | **Myc** | 84% | 100% | 99% | 0 | 0 | 0 |
| lysosomal alpha-mannosidase-like | gi\|357156617 | 113 kDa | **Myc** | 65% | 100% | 100% | 0 | 0 | 0 |
| nitrile-specifier protein 5 | gi\|225447524 | 35 kDa | **Myc** | 100% | 100% | 100% | 0 | 0 | 0 |
| hypothetical protein | gi\|300138377 | 80 kDa | **Myc** | 85% | 100% | 95% | 0 | 0 | 0 |
| oryzain beta chain | gi\|109939735 | 50 kDa | **Myc** | 100% | 99% | 100% | 0 | 0 | 0 |
| LRR receptor-like ser/thr-protein kinase * | gi\|125581346 | 111 kDa | **Myc** | 85% | 81% | 98% | 0 | 0 | 0 |
| vacuolar 69 kDa subunit | gi\|1049253 | 62 kDa | **Myc** | 99% | 53% | 100% | 0 | 0 | 0 |
| glyceraldehyde-3-phosphate dehydrogenase 1 | gi\|258642941 | 37 kDa | **Myc** | 47% | 97% | 100% | 0 | 0 | 0 |
| arabinosidase ARA-1 | gi\|16417958 | 74 kDa | **Myc** | 100% | 100% | 0 | 0 | 0 | 0 |
| RUBISCO large subunit | gi\|156454192 | 51 kDa | **Myc** | 95% | 96% | 0 | 0 | 0 | 0 |
| hypothetical protein ARALYDRAFT_354856 | gi\|297313807 | 12 kDa | **Myc** | 68% | 99% | 75% | 0 | 0 | 0 |
| Aspartic proteinase* | gi\|78099760 | 54 kDa | **Myc** | 96% | 97% | 11% | 0 | 0 | 0 |
| predicted protein | gi\|162662890 | 31 kDa | **Myc** | 100% | 0 | 83% | 0 | 0 | 0 |
| cucumisin* | gi\|297321645 | 40 kDa | **Myc** | 96% | 42% | 0 | 0 | 0 | 0 |
| actin-1, putative, expressed | gi\|108710772 | 42 kDa | **Myc** | 100% | 100% | 100% | 0 | 0 | 0 |
| hypo. SORBIDRAFT_09g019152 | gi\|241946316 | 32 kDa | **Myc** | 99% | 98% | 0 | 0 | 0 | 0 |
| ATPase alpha subunit (mitochondrion) | gi\|388890762 | 55 kDa | **Myc** | 0 | 76% | 99% | 0 | 0 | 0 |
| eukaryotic translation initiation factor 3 | gi\|356524672 | 105 kDa | **Myc** | 0 | 100% | 70% | 0 | 0 | 0 |
| glucan endo-1,3-beta-glucosidase 11 | gi\|75154301 | 35 kDa | **Myc** | 64% | 99% | 0 | 0 | 0 | 0 |
| ATP synthase alpha subunit | gi\|20146556 | 43 kDa | **Myc** | 100% | 0 | 100% | 0 | 0 | 0 |
| cysteine proteinase | gi\|595986 | 47 kDa | **Myc** | 0 | 92% | 100% | 0 | 0 | 0 |
| putative plasma membrane intrinsic protein | gi\|27527694 | 31 kDa | **Myc** | 98% | 0 | 83% | 0 | 0 | 0 |
| patellin-1* | gi\|78099065 | 17 kDa | **Myc** | 0 | 79% | 98% | 0 | 0 | 0 |
| disease resistance protein RPP13* | gi\|29839653 | 105 kDa | **Myc** | 47% | 95% | 0 | 0 | 0 | 0 |
| hypothetical protein VOLCADRAFT_105607 | gi\|300262036 | 330 kDa | **Myc** | 0 | 98% | 42% | 0 | 0 | 0 |
| glutathione reductase | gi\|1370285 | 54 kDa | **6.7** | 0 | 100% | 100% | 100% | 100% | 0 |
| cytosolic ascorbate peroxidase | gi\|120969450 | 27 kDa | **5.5** | 45% | 100% | 96% | 100% | 0 | 0 |
| predicted protein | gi\|326502400 | 59 kDa | **5.3** | 0 | 100% | 100% | 100% | 0 | 52% |
| fructose-bisphosphate aldolase | gi\|226316441 | 39 kDa | **5.2** | 96% | 96% | 99% | 84% | 99% | 90% |
| vacuolar H+-pyrophosphatase | gi\|336112676 | 80 kDa | **5** | 0 | 0 | 100% | 51% | 100% | 0 |
| aquaporin | gi\|1212923 | 25 kDa | 3.2 | 100% | 100% | 100% | 100% | 100% | 0 |
| NADP malic enzyme4 | gi\|162463047 | 72 kDa | 3 | 100% | 0 | 0 | 76% | 0 | 95% |
| glyceraldehyde-3-phosphate dehydrogenase | gi\|120669 | 37 kDa | 2.9 | 100% | 99% | 99% | 99% | 99% | 100% |
| pathogenesis-related protein | gi\|359840826 | 17 kDa | 2.9 | 96% | 64% | 97% | 66% | 42% | 99% |
| ATPase subunit 1 (mitochondrion) | gi\|340549482 | 55 kDa | 2.6 | 11% | 100% | 100% | 100% | 0 | 100% |
| pyruvate kinase* | gi\|2497538 | 57 kDa | 2.5 | 100% | 100% | 100% | 100% | 100% | 100% |
| elongation factor EF-2 | gi\|355485214 | 94 kDa | 2.5 | 0 | 100% | 100% | 100% | 100% | 100% |
| core histone H2A/H2B/H3/H4 family protein | gi\|108707495 | 44 kDa | 2.3 | 100% | 100% | 100% | 100% | 100% | 100% |
| enolase | gi\|1169534 | 48 kDa | 2.2 | 100% | 100% | 100% | 100% | 100% | 100% |
| glyceraldehyde-3-phosphate dehydrogenase | gi\|462137 | 37 kDa | 2.1 | 100% | 100% | 100% | 100% | 100% | 100% |
| elongation factor 1-alpha | gi\|327164449 | 49 kDa | -2 | 99% | 100% | 0 | 100% | 100% | 100% |
| malate dehydrogenase* | gi\|18202485 | 36 kDa | -2 | 0 | 100% | 100% | 100% | 100% | 100% |
| elongation factor 2* | gi\|6015065 | 94 kDa | -2,5 | 100% | 0 | 100% | 100% | 100% | 100% |
| heat shock protein 90 | gi\|110270498 | 80 kDa | -3,3 | 100% | 0 | 0 | 100% | 80% | 96% |
| Enolase | gi\|1169534 | 48 kDa | **-5** | 100% | 0 | 5% | 100% | 100% | 100% |
| homocysteine methyltransferase* | gi\|8134566 | 85 kDa | **-5** | 100% | 100% | 100% | 100% | 100% | 100% |
| chaperonin CPN60-2 | gi\|355480690 | 61 kDa | **-5** | 100% | 0 | 0 | 100% | 100% | 100% |
| heat shock protein 70 | gi\|189380223 | 75 kDa | **-5** | 100% | 100% | 100% | 100% | 100% | 100% |
| lipoxygenase, partial | gi\|384407029 | 49 kDa | **Non-myc** | 0 | 0 | 0 | 100% | 100% | 100% |
| methionine synthase protein, partial | gi\|374256013 | 33 kDa | **Non-myc** | 0 | 0 | 0 | 100% | 100% | 100% |
| FLORICAULA/LEAFY | gi\|67515298 | 6 kDa | **Non-myc** | 66% | 21% | 5% | 100% | 95% | 96% |
| alpha-tubulin | gi\|134035494 | 50 kDa | **Non-myc** | 0 | 0 | 0 | 100% | 100% | 100% |
| homocysteine methyltransferase, putative | gi\|223535009 | 85 kDa | **Non-myc** | 0 | 0 | 0 | 100% | 100% | 100% |
| L-ascorbate peroxidase 1* | gi\|122247194 | 24 kDa | **Non-myc** | 0 | 0 | 76% | 100% | 100% | 100% |
| subtilisin-like protease SDD1 | gi\|75099062 | 79 kDa | **Non-myc** | 0 | 0 | 0 | 100% | 100% | 100% |
| lypoxygenase | gi\|165928943 | 39 kDa | **Non-myc** | 0 | 0 | 0 | 100% | 100% | 100% |
| hypothetical protein VITISV_041925 | gi\|147809484 | 26 kDa | **Non-myc** | 0 | 0 | 0 | 100% | 100% | 100% |
| malate dehydrogenase | gi\|344190166 | 36 kDa | **Non-myc** | 0 | 0 | 0 | 100% | 100% | 100% |
| 60S ribosomal protein L3-1* | gi\|27735225 | 63 kDa | **Non-myc** | 0 | 0 | 0 | 98% | 100% | 100% |
| conserved hypothetical protein | gi\|223519414 | 8 kDa | **Non-myc** | 0 | 0 | 0 | 100% | 100% | 93% |
| fructokinase | gi\|1052973 | 35 kDa | **Non-myc** | 0 | 0 | 0 | 100% | 100% | 0 |
| fructose-bisphosphate aldolase* | gi\|1168410 | 28 kDa | **Non-myc** | 0 | 0 | 0 | 100% | 98% | 0 |
| GDP-mannose 3',5'-epimerase | gi\|110740559 | 43 kDa | **Non-myc** | 0 | 0 | 0 | 97% | 100% | 100% |
| 60S ribosomal protein L11* | gi\|1173055 | 20 kDa | **Non-myc** | 54% | 0 | 0 | 100% | 100% | 0 |
| allene oxide synthase 3* | gi\|73619654 | 54 kDa | **Non-myc** | 0 | 0 | 0 | 100% | 100% | 0 |
| S-adenosylmethionine synthetase | gi\|100801600 | 43 kDa | **Non-myc** | 0 | 0 | 0 | 100% | 100% | 0 |
| clathrin heavy chain 1* | gi\|122223702 | 193 kDa | **Non-myc** | 0 | 0 | 0 | 100% | 100% | 0 |
| 26S proteasome subunit 11* | gi\|75264101 | 46 kDa | **Non-myc** | 0 | 0 | 0 | 0 | 100% | 97% |
| adenine nucleotide translocator | gi\|1297066 | 36 kDa | **Non-myc** | 0 | 83% | 0 | 0 | 99% | 98% |
| elongation factor 1-alpha* | gi\|6015058 | 49 kDa | **Non-myc** | 44% | 0 | 0 | 0 | 100% | 100% |
| phosphoglycerate kinase, putative | gi\|102139814 | 42 kDa | **Non-myc** | 0 | 0 | 0 | 100% | 0 | 100% |
| peroxidase 51* | gi\|26397925 | 36 kDa | **Non-myc** | 0 | 0 | 0 | 100% | 0 | 100% |
| ubiquitin monomer protein | gi\|73747822 | 17 kDa | **Non-myc** | 0 | 0 | 0 | 100% | 100% | 0 |
| allene oxide synthase | gi\|375004669 | 45 kDa | **Non-myc** | 0 | 0 | 0 | 99% | 100% | 0 |
| PREDICTED: RING-H2 finger protein | gi\|357127651 | 23 kDa | **Non-myc** | 0 | 0 | 0 | 49% | 99% | 0 |
| isocitrate dehydrogenase* | gi\|1708402 | 47 kDa | **Non-myc** | 0 | 0 | 0 | 28% | 100% | 0 |
| 40S ribosomal protein S14* | gi\|131772 | 16 kDa | **Non-myc** | 0 | 0 | 0 | 0 | 100% | 93% |
| pentatricopeptide repeat-containing protein | gi\|297336922 | 63 kDa | **Non-myc** | 0 | 0 | 0 | 87% | 95% | 0 |

FC =Fold change in relation to the reference category; MW= Predicted molecular weight. Myc1, Myc2 and Myc3 values refers to the protein identification probability in each biological replicate. Ref refers to values in reference samples (in this case, non-mycorrhizal roots). Myc and Non-Myc (in the FC columns) refers to proteins which are assumed to be only accumulated in mycorrhizal or non-mycorrhizal roots respectively. * pBLAST best hit with e-value < 10e-5. Fold change values written in bold are statistically significant (*p* < 0.05).

**Table S4.** Relative quantification of proteins with significant differential accumulation in mycorrhizal and non-mycorrhizal roots of *Oeceoclades maculata* using iTRAQ

| **Highest hit in database** | **Accession Number** | **MW Test** | **S1-R** | **S2-F** | **S2-R** | **S3-F** | **S3-R** | **Average** | **FC** |
| --- | --- | --- | --- | --- | --- | --- | --- | --- | --- |
| 40S ribosomal protein S15 [*Arabidopsis thaliana*] | gi\|10178203 | 95% (0.021) | 0 | 1.6 | 1 | 1.1 | 1.9 | 1.12 | 2.2 |
| PR protein [*Musa acuminata* AAA Group] | gi\|359840826 | 95% (0.0090) | 0.6 | 0.7 | 0.8 | 1 | 0.9 | 0.8 | 1.7 |
| peroxidase 12-like [*Brachypodium distachyon*] | gi\|357157932 | 95% (0.021) | 0.3 | 0 | 0.4 | 1.4 | 1.3 | 0.68 | 1.6 |
| SORBIDRAFT_04g019020 [*Sorghum bicolor*] | gi\|241933653 | 95% (0.0017) | - | 0.5 | 0.8 | 0.8 | 0.5 | 0.65 | 1.6 |
| predicted protein [*Hordeum vulgare* subsp. *vulgare*] | gi\|326527391 | 95% (0.00016) | 1 | 0.4 | 0.7 | 0.4 | 0.5 | 0.6 | 1.5 |
| lypoxygenase [*Capsicum annuum*] | gi\|165928943 | 95% (0.0090) | -0.8 | -0.6 | -0.5 | -0.5 | -0.5 | -0.58 | -1.5 |
| SELMODRAFT_129301 [*Selaginella moellendorffii*] | gi\|300143068 | 95% (0.0090) | -1 | -1 | -0.8 | -0.2 | -0.2 | -0.64 | -1.6 |
| PREDICTED: annexin D3-like [*Glycine m*ax] | gi\|356556843 | 95% (0.0090) | -0.9 | -0.5 | -0.3 | -0.6 | -1 | -0.66 | -1.6 |
| heat shock protein [*Fragaria* x *ananassa*] | gi\|54306589 | 95% (0.021) | 0 | -1.3 | -1.2 | -0.4 | -0.5 | -0.68 | -1.6 |
| 16.9 kDa class I heat shock protein 1 [*Zea mays*] | gi\|195605946 | 95% (0.021) | -2.2 | -1.3 | -1.5 | - | - | -1.66 | -3.2 |

S1-F, S2-F and S3-F: biological sample replicates 1, 2 and 3 with forward tagging; S1-R, S2-R and S3-R: biological sample replicates 1, 2 and 3 with reverse tagging. FC= Fold change. MW test = Mann Whittney test (*p*-value). Values from each replicate and Average are expressed as log_2_.

**Table S5:** Statistics of cDNA sequences derived from *Oeceoclades maculata* roots

| **Sample^1^** | **Total reads** | **Total reads after merging^2^** | **% trimmed** | **Number of reads completely removed** | **Avg. lenght before merging (bp)** | **Avg. lenght after merging (bp)** | **Avg. lenght after trimming (bp)** |
| --- | --- | --- | --- | --- | --- | --- | --- |
| M1 | 13,258,869 | 18,843,992 | 0.9980 | 50,334 (0,26%) | 101 | 150 | 147 |
| M2 | 15,768,946 | 21,089,332 | 0.9985 | 56,111 (0,26%) | 101 | 148 | 145 |
| M3 | 14,149,071 | 18,921,006 | 0.9965 | 55,806 (0,29%) | 101 | 148 | 144 |
| NM1 | 14,526,535 | 19,241,370 | 0.9975 | 53,539 (0,27%) | 101 | 148 | 144 |
| NM2 | 12,992,840 | 17,001,980 | 0.9995 | 48,969 (0,28%) | 101 | 147 | 144 |
| NM3 | 12,631,275 | 16,334,516 | 0.9996 | 42,959 (0,26%) | 101 | 145 | 142 |
| Total | 83,354,536 | 111,432,196 |  |  |  |  |  |

^1^ M= Mycorrhizal root; NM= Non-Mycorrhizal root. ^2^Sum of non-merged + merged reads

**Table S6:** Length distribution of *de novo* assembled contigs.

| **Parameter^1^** | **Base pairs** |
| --- | --- |
| N75 | 471 |
| N50 | 1066 |
| N25 | 2069 |
| Minimum length | 120 |
| Maximum length | 16788 |
| Average length | 717 |

^1^ N75 is the point where 75% of the entire assembly is contained contigs equal o larger than this value. Accordingly, N50 is the point of half of the mass of the contig length distribution and N25 is the point where 25% of the assembly is contained in contigs equal to or larger than this value. A total of 81,666 contigs was obtained.

| 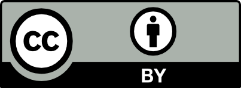 | © 2020 by the authors. Submitted for possible open access publication under the terms and conditions of the Creative Commons Attribution (CC BY) license (http://creativecommons.org/licenses/by/4.0/). |
| --- | --- |
